# Beliefs About Causes and Cures of Prolonged Grief Disorder Among Arab and Sub-Saharan African Refugees

**DOI:** 10.3389/fpsyt.2022.852714

**Published:** 2022-04-05

**Authors:** Franziska Lechner-Meichsner, Hannah Comtesse

**Affiliations:** ^1^Clinical Psychology and Psychotherapy, Department of Psychology, Goethe University Frankfurt, Frankfurt, Germany; ^2^Clinical and Biological Psychology, Department of Psychology, Catholic University Eichstaett-Ingolstadt, Eichstaett, Germany

**Keywords:** prolonged grief disorder (PGD), refugees, traumatic loss, help-seeking, beliefs, mental health, qualitative content analysis

## Abstract

**Background:**

Many refugees have experienced the death of a loved one under traumatic circumstances. Accordingly, the prevalence of Prolonged Grief Disorder (PGD) among refugees is high. Culture-specific symptoms of PGD have been described previously, but beliefs about causes and cures of PGD among refugees remain unknown. We therefore aimed at identifying illness beliefs and treatment expectations regarding PGD among refugees.

**Method:**

We focused on refugees from Arab countries (*n* = 14) and from Sub-Sahara Africa (*n* = 9) and applied qualitative and quantitative methods. In a semi-structured interview, participants first answered questions about assumed causes and potential cures for prototypical PGD symptoms according to ICD-11 that were presented in a vignette as representatives of their own culture. In the quantitative part, they completed the Cause Subscale of the Illness Perception Questionnaire (IPQ-R) that included additional culture-specific items. Interviews were analyzed with Qualitative Content Analysis.

**Results:**

In both groups of refugees, PGD symptoms were predominantly attributed to a close relationship to the deceased, lack of social support, personal vulnerabilities, and circumstances of the death. Participants also named a number of flight-related causes (e.g., inability to perform or participate in rituals, feeling isolated in the host country). None of the participants attributed PGD symptoms to supernatural causes. Descriptive analyses of responses on the IPQ-R indicated that participants predominantly attributed PGD symptoms to psychological causes. Participants believed that PGD can be cured and predominantly mentioned social and religious support. Psychological help was only mentioned by a minority of participants. In both groups, participants emphasized that a therapist must be familiar with the patient's culture and rituals. Participants also mentioned stigma associated with seeking psychological help.

**Conclusion:**

Results suggest specific beliefs of refugees regarding causes and cures of PGD as well as similarities with Western conceptualizations. A culture-sensitive approach to the treatment of PGD in refugees that can include knowledge of culture-specific rituals and incorporating religious beliefs as well as decreasing stigma and increasing mental health literacy seem important. The study is limited by its focus on only two groups of refugees and its small sample size.

## Introduction

In the past 10 years, at least 100 million people worldwide have been displaced. With more than 2 million asylum applications, Germany recorded the highest number of applications in Europe during this period (1). Most refugees come from Syria, Afghanistan, Iraq, and countries of Sub-Sahara Africa such as Somalia or Eritrea (2). Many refugees report traumatic experiences and deaths of loved ones are among the most frequently reported events [e.g., (3)]. Accordingly, the prevalence of prolonged grief disorder (PGD) in refugee samples is high [e.g., (4, 5)]. PGD is a new diagnosis in ICD-11 and DSM-5-TR that is characterized by persistent and pervasive yearning for a deceased person and/or pre-occupation with this person that persist for an uncharacteristically long time (>6 months in ICD-11; ≥12 months in DSM-5-TR) (6, 7). Bryant et al. (8, 9) reported PGD prevalence rates of 15.8 and 15.1% in representative samples of bereaved refugees in Australia and Jordan, respectively. These numbers are about three times higher than the numbers from representative samples of bereaved Westerners after mostly non-violent loss [e.g., (10)]. However, the percentage of those refugees who receive adequate support for their mental-health problems is generally low (11, 12) and refugees are less likely to seek or be referred to mental health services (13–15). Given the prevalence of PGD, the high number of refugees in Western countries, and the general treatment gap, there seems to be a need to adapt grief-focused treatments to refugees' specific needs.

Although there is growing evidence of the cross-cultural validity of the PGD construct and its measures (16, 17), the expression of grief may differ within the respective cultural and religious frameworks (18, 19). A systematic review on the assessment of PGD in refugee and post-conflict samples (18) has shown that PGD can be identified with measures developed with Western samples. However, the few studies that used culturally adapted measures, revealed culture-specific symptoms of grief: somatic complaints, spiritual concerns, and dreams of or re-experiencing the deceased person were common expressions of impairing grief. These symptoms were highly prevalent and not captured by standard measures, thus pointing to the need of culture-sensitive assessment of PGD. A recent qualitative study with Syrian refugees identified emotional outbursts and weariness as additional symptoms (20). Furthermore, there is growing evidence that the conceptualization of mental disorders differs between cultural groups (21). Cultural barriers could therefore hamper the treatment of bereaved refugees in Western countries. To enhance the acceptability and effectiveness of psychological interventions for refugees, the cultural adaptation framework (21) suggests to systematically investigate the relevance of adapting treatment to (a) cultural concepts of distress, (b) treatment components, and (c) treatment delivery. It has also been suggested that treatments for refugees should promote established mechanisms of recovery in a culture-sensitive way and tailor treatments to patients' individual situations and life histories in order to reduce barriers (22). A recent uncontrolled study of grief-focused cognitive-behavioral therapy (CBT), that was integrated in a day-patient treatment program for refugees in the Netherlands, led to a large reduction in grief symptoms (23). Grief-focused CBT has been shown to be highly efficient in the treatment of PGD in patients from mostly Western countries (24) and the results by Djetlantik et al. (25) point toward the general effectiveness of common treatment elements for PGD (e.g., exposure to avoided stimuli or memories) in refugees. In addition, it has been argued that CBT programs for the treatment of refugees benefit from adaptations regarding content or structure [e.g., group setting, appropriate metaphors; (22, 26, 27)] that make programs sensitive for the target population's needs. To be able to make such culture-sensitive adaptations to grief-focused CBT, it would be an important starting point to learn more about refugees' illness beliefs and treatment expectations regarding PGD.

Several studies investigated lay beliefs about mental disorders among refugees in Western countries. A Somali sample distinguished three major types of mental problems: sadness or suffering (“murug”), craziness due to spirit possession (“gini”), and craziness due to severe trauma (“waali”) (28). Grupp et al. (29) interviewed asylum seekers from Sub-Sahara Africa in focus groups about their beliefs regarding causes behind symptoms of post-traumatic stress disorder (PTSD) and identified traumatic life experiences, psychological causes, social causes, post-migration stressors, religious causes, and supernatural causes. In a larger survey part of the study, asylum seekers attributed symptoms more strongly to religious and supernatural causes than German participants without a migration background. Other studies also found that lay explanatory models of Sub-Saharan refugees emphasize supernatural influences or God's will (30, 31). Causes for depression were also seen in a person's social situation, especially being lonely and away from family, and the emotional reaction to difficult life (31).

Causal beliefs influence treatment-seeking and health care utilization (31). The perceived quality of health care can also be diminished by divergent expectations and frustrations can arise on both sides (32). Sub-Saharan refugees tend to seek help from religious treatments including prayer and religious authorities (28, 30, 33, 34) and traditional treatments such as herbal teas, natural remedies, or offerings (28, 34). Social support also plays a major role and Sub-Saharan refugees prefer to utilize the support from family and friends (28, 31, 33–35). Studies have shown that refugees from African countries are less likely to seek help from mental health professionals than people without a migration background (34) und under-utilize the available services (12, 33, 36) due to fear of stigmatization and lack of knowledge and accessibility (33). In an investigation with Syrian refugees in Germany, social support was also mentioned as one of the most important sources of support (37). While participants also mentioned seeking help from psychologists, there was no clear understanding of how mental health professionals work. Syrian refugees also reported more negative attitudes toward professional help than German residents despite higher levels of depressive symptoms and functional impairment that was mediated by lower tendency to disclose personal information and distress (38). Again, common barriers to utilizing help from mental health professionals were lack of information, stigma and language problems (37, 39).

Taken together, refugees from non-Western cultural groups tend to attribute mental health problems more to religious or spiritual causes or situational factors than to medical models behind Western treatment programs. Accordingly, they are likely to seek cures in religious and traditional treatments as well as social support. Mental health services are known, but fear of stigmatization and lack of knowledge are often barriers to their utilization. Although these findings provide valuable insights into how interventions can be tailored for refugees, they are based on investigations on mental health problems in general, or depression or PTSD and it is unknown if the same beliefs apply to PGD. Opinions about grief as a mental disorder have been explored in different cultures [e.g., (40, 41)], but perceived causes or treatment expectations have not been studied yet. The present study therefore aims to examine illness beliefs and treatment expectations of refugees regarding symptoms of PGD for the first time. We focused on refugees from Arab and Sub-Sahara African countries because they constitute important refugee groups in Germany. In Arab countries in Western Asia, Northern Africa, Western Africa, and Eastern Africa most people adhere to Islam that also shapes rituals and expectations surrounding grief. A 3-day funeral service attended by often large groups of extended family, friends, neighbors, and acquaintances is common during which emotions are expressed through crying or singing. This is followed by a 40-day mourning period for the family that ends by holding a funeral meal. During this time, support is offered to the family of the deceased. The term Sub-Sahara Africa is used for the group of countries that are located fully or partially south of the Sahara and that are heterogeneous regarding languages, ethnic groups, and religion. Christianity is the most common faith, followed by Islam, and traditional African religions that generally include animism and ancestor worship.^1^

We applied a mixed methods strategy by complementing a structured interview on beliefs about causes of PGD symptoms and treatment expectations with a questionnaire on the perceived causes of PGD symptoms. This combination enabled us to explore subjective meanings as well as theory-driven constructs.

## Methods

### Procedure

A convenience sample was recruited between February and April 2020 *via* social media, word-of-mouth, and printed ads in places frequented by refugees. Target sample size was based on similar studies in this field [e.g., (28, 30)]. Inclusion criteria were being at least 18 years old and having come to Germany as a refugee from an Arab or a Sub-Saharan country. When potential participants contacted the study personnel, they received written and oral information on the study's aims and procedure. After providing informed consent, participation consisted of two parts: First, a semi-structured interview was conducted. At the beginning of the interview, participants were presented with a vignette that described a person with prototypical PGD symptoms. Interviews lasted about 60 min and at the end, participants received a link to an online survey where data about illness perception, loss history and own PGD symptoms, and sociodemographic information were collected. The survey took 20–30 min to complete and was available in German, English, and Arabic. Interviews were conducted with the help of an interpreter when necessary. Participants received a voucher for 10 Euros for their participation in the study. One person declined participation due to problems scheduling an appointment for the interview. The study was approved by the ethics committee of the Goethe University Frankfurt (reference numbers 2020-20 and 2019-57).

### Materials and Measures

#### Vignette

A standardized vignette described a person with PGD symptoms according to ICD-11 and was presented at the beginning of the interview and the online survey. The vignette was adapted from a vignette of PGD symptoms presented in a study with a German sample without migration background (42). Assault was chosen as the cause of death because many refugees have experienced traumatic loss (23). The gender of the described person was matched to the participant's gender. The vignette was read aloud by the interviewer and shared via the computer screen and it was presented at the first page of the online survey. It read as follows:

*A. is 35 years old and has been living in Germany for two years. Three years ago, her mother was assaulted in her home country and died. A. still cannot comprehend that her mother has died and has trouble fulfilling her everyday tasks. She is yearning for her mother and feels strong grief and intense emotional pain every day. She is thinking about her mother all the time. She ruminates about the circumstances of the death and asks herself if her mother was in pain and why it was her who died. She feels bitter and cannot accept the loss. She has strong feelings of guilt, because she was not with her mother and could not help her. She avoids things that remind her of her mother, such as telephone calls with her two siblings or news from her home town, because she fears that the pain might become too strong. Instead, she often looks at pictures of her mother because this makes her feel close to her. She feels she lost a part of herself and doesn't know who she is anymore without her mother and how her life should go on. Positive feelings are rare, instead she is angry and sad most of the time. A. has withdrawn from other people*.

#### Interview

Interviews followed a semi-structured guideline (43) that was derived from the research questions and informed by the Cultural Formulation Interview in DSM-5 (44). In the beginning, the interviewer introduced herself, reviewed the goals of the study, and asked participants to answer questions as a representative of their home country or cultural group. She then asked questions about four main topics: (a) perception of pathological grief symptoms, (b) duration of grief, (c) beliefs about causes, (d) beliefs about cures (see Supplementary Data Sheet 1). If necessary, probes were given to further explain or elaborate on answers. For the present study, only responses regarding causes and cures were analyzed. The interviewer first asked participants to answer regarding the vignette and then to add anything they perceive important for grieving persons in general (e.g., “What do you think caused the described person's prolonged and impairing grief?;” “Do you think that prolonged grief disorder can be cured in general?”). Interviews were conducted by two female Master-level students in psychology who were trained in conducting the interviews by the first author. As the time of the data collection coincided with the COVID-19 pandemic and physical distancing regulations were in place, interviews were performed via a videoconferencing software. Participants were asked to select a private quiet place for the interview, interviewers were alone in a room when conducting the interviews. All interviews were recorded.

#### Illness Perceptions

Quantitative assessment of perceived causes of PGD symptoms was conducted with the Illness Perception Questionnaire–Revised [IPQ-R; (45)]. Only the subscale that captures causal assumptions of diseases was used. The original scale consists of 18 items (e.g., “personality,” or “family problems”). For reasons of cultural sensitivity, three additional items (“God's will,” “evil spirits,” and “supernatural forces”) were added based on previous research with non-Western samples (46, 47).

The original instruction was modified and participants were asked to indicate how likely they perceived each cause with regard to the person described in the vignette. Agreement was rated on a scale from 1 (strongly disagree) to 5 (strongly agree). The IPQ-R has demonstrated satisfying reliability and validity in previous studies (45, 47) and was also used to assess beliefs regarding symptoms presented in a vignette (29). IPQ-R versions were available in all three study languages (45, 48, 49).

### PGD Symptoms and Interpersonal Loss History

Participant's own interpersonal loss history was assessed with items regarding how many members of the family and close friends had died (see Supplementary Data Sheet 2). If participants had experiences losses, the 18-item Traumatic Grief Inventory Self-Report Version [TGI-SR; (50)] was used to assess PGD symptom severity. Participants were asked to consider their most distressing loss and rate the extent to which they experienced grief symptoms during the last month on a 5-point scale from 1 (never) to 5 (always). A total PGD symptom severity score (range: 18–90) was calculated. The TGI-SR is a reliable and valid instrument for the measurement of PGD symptoms and has been used previously with refugee samples [e.g., (51)].

### Sociodemographic Information

Sociodemographic characteristics and migration-related information (e.g., gender, age, country of birth, time since arrival in Germany; see Supplementary Data Sheet 2) were collected at the end of the survey.

### Analysis

#### Qualitative Analysis

Reporting of the qualitative analysis and results is guided by the COREQ criteria [(52); Supplementary Data Sheet 3]. Interview recordings were transcribed verbatim and analyzed with the software MaxQDA using qualitative content analysis (53, 54). This systematic, rule-guided method of text analysis allows to analyze large quantities of text material and structure it according to a specific research question. The analysis follows a set of previously established steps, the most important being the development of a *category system*. This is a coding scheme that can be developed in an inductive (i.e., abstracting categories from the text) or deductive (i.e., developing categories based on theoretical principles) way. For each category, a category system includes a definition, coding rule, and anchor items (i.e., good examples of the category). With the help of the category system, categories are then assigned to previously defined coding units. To answer research questions pertaining to beliefs regarding causes and cures for PGD, one category system for each was developed inductively. Coding units were defined as paragraphs where participants answered questions regarding causes or cures or where they explicitly referred to causes or cures elsewhere during the interview.

The analysis was conducted in five steps. First, both authors read through all transcripts. Second, the first author (FLM) re-read the transcripts and started to develop categories that summarize causes and cures mentioned by the participants. After one third of the transcripts, the categories were checked for a comparable level of abstraction and category definitions were developed. Related categories were organized under superordinate categories. Coded paragraphs were checked for their fit with the categories. The first author then worked through the remaining number of transcripts assigning units to already existing categories and creating new categories when novel aspects emerged. After all transcripts were coded, she reviewed the category names and definitions. Third, the second author (HC) reviewed the category systems and coded units. Fourth, discrepancies were discussed and joint decisions made for the final code assignment. Frequencies for each category were calculated in the last step.

FLM is experienced in conducting qualitative research. Both FLM and HC have experience in working with refugees in research and clinical practice settings.

#### Quantitative Analyses

Responses on the IPQ-R and demographic characteristics were analyzed using SPSS 28. The IPQ-R response categories “agree” and “strongly agree” were combined into one broader agreement category. Absolute and relative frequencies of agreement with each of the 21 items were then computed for the total sample, the Arab sample and the Sub-Saharan sample. Due to the small number of participants in each of the two groups (*n* = 14 and *n* = 9, respectively), no tests for statistical significance were performed.

## Results

### Sample

The sample comprised 23 participants. The Arab sample was slightly larger (*n* = 14) than the Sub-Saharan African sample (*n* = 9). Mean time since arrival in Germany was 5.5 years (*SD* = 6.8) in the Arab sample and 12.6 years (*SD* = 12.4) in the Sub-Saharan African sample. A detailed description of sociodemographic and migration-related characteristics is presented in Table 1.

**Table 1:** Sociodemographic, migration-, and loss-related characteristics.

**Characteristic**	**Total sample** **(*N* = 23)**	**Participants from Arab countries** **(*n* = 14)**	**Participants from Sub-Sahara Africa** **(*n* = 9)**
Female, % (*n*)	56.5% (13)	50.0% (9)	44.0% (4)
Age in years, *M* (*SD*)	29.7 (14.6)	24.1 (10.3)	38.5 (16.5)
**Country of birth, % (** ***n*** **)**
Syria	71.5% (10)	71.5% (10)	–
Yemen	14.3% (2)	14.3% (2)	–
Iraq	7.1% (1)	7.1% (1)	–
Egypt	7.1% (1)	7.1% (1)	–
Cameroon	11.1% (1)	–	11.1% (1)
Côte d'Ivoire	11.1% (1)	–	11.1% (1)
Congo	11.1% (1)	–	11.1% (1)
Eritrea	11.1% (1)	–	11.1% (1)
Ethiopia	11.1% (1)	–	11.1% (1)
Gambia	11.1% (1)	–	11.1% (1)
Ghana	11.1% (1)	–	11.1% (1)
Guinea	11.1% (1)	–	11.1% (1)
Somalia	11.1% (1)	–	11.1% (1)
**Religion, % (** ***n*** **)**
Islam	65.2% (15)	92.9% (13)	22.2% (2)
Christianity	26.1% (6)	0.0% (0)	66.7% (6)
Not available	8.7% (2)	7.1% (1)	11.1% (1)
Time since arrival in Germany in years, *M* (*SD*)	8.3 (9.8)	5.5 (6.8)	12.6 (12.4)
Number of losses^a^, *M* (*SD*)	1.8 (1.4)	1.5 (0.9)	2.4 (1.9)
Prolonged grief symptoms^b^, *M* (*SD*)	34.3 (11.5)	32.4 (11.8)	38.5 (10.7)

*–, not available*.

a *Losses of significant persons were assessed with a loss questionnaire [see Comtesse and Rosner (4)]*.

b *Symptoms of prolonged grief were assessed with the TGI-SR (50)*.

Arab participants had experienced a mean number of 1.5 losses (*SD* = 0.9), Sub-Saharan African participants had experienced 2.4 losses (*SD* = 1.9) on average. None of the participants scored above the cut-off for PGD as measured with the TGI-SR. In response to the vignette, most participants described the PGD symptoms as familiar and many gave examples from people they knew. One participant (woman 2, Syria) spoke about her intense grief after the loss of her father.

### Causes

#### Qualitative Results

The causes participants believed to be responsible for the symptoms, were grouped into four superordinate categories: (1) interpersonal causes, (2) intrapersonal causes, (3) circumstances of the death, and (4) separation from home and loved ones (Table 2).

**Table 2:** Causes of PGD symptoms.

	**Total sample (*****N*** **=** **23)**		**Participants from Arab**		**Participants from Sub-Sahara**	
			**countries (*****n*** **=** **14)**		**Africa (*****n*** **=** **9)**	
**Cause**	***N***	**%**	***N***	**%**	***N***	**%**
**Separation from home and loved ones**	7	30.4	4	28.6	3	33.3
**Interpersonal**						
Emotional bond	15	65.2	11	78.6	4	44.4
Kinship	11	47.8	8	57.1	3	33.3
Lack of social support	10	43.5	6	42.9	4	44.4
Material dependency	4	17.4	2	14.3	2	22.2
Aggravation by others	1	4.3	1	7.1	0	0.0
**Intrapersonal**						
Vulnerability	12	52.2	8	57.1	4	44.4
Guilt	7	30.4	6	42.9	1	11.1
Pre-existing mental health problems or traumatic experiences	3	13.0	2	14.3	1	11.1
Religious beliefs	1	4.3	1	7.1	0	0.0
**Circumstances of the death**						
Violent or unnatural death	8	34.8	7	50.0	1	11.1
Sudden death	7	30.4	6	42.9	1	11.1
Unable to perform rituals	6	26.1	2	14.3	4	44.4
Loved one died alone	1	4.3	1	7.1	0	0.0
Multiple losses	1	4.3	1	7.1	0	0.0

*PGD, prolonged grief disorder*.

##### Interpersonal Causes

Interpersonal causes were mentioned by 91.3% of participants and five sub-categories emerged.

###### Emotional Bond (N = 15).

The category “emotional bond” was assigned when participants indicated that PGD symptoms develop because the bereaved person shared a close emotional bond with the deceased that was unique compared to their other relationships.

*My cousin, when she lost her mom… you know her mom was her everything. And in the example, I don't know, if she [also] took her mom as her everything. So when [my cousin] lost [her] mother, she can never find anyone like her mother again, do you get that? She can never never find somebody like her mom. (woman 10, Ghana)* ^2^

Participants described strong bonds among family members, especially among parents, children, and spouses but also among friends.

*Our families are very big, we have a lot of children, you know? But you only have one best friend, maybe two. And when one of them is not here anymore, you feel very lonely. (man 1, Syria)*

*When I am only connected to one person and when this person cannot give me air anymore, then I'll die and when this person dies, I will die as well. Everything in my life is because of that person and not because of me. My luck is tied to him and when he is gone, I am also out of luck. (man 2, Syria)*

A strong emotional bond as a cause of PGD was more often mentioned by refugees from Arab countries.

###### Kinship (N = 11).

The category “kinship” comprises statements where participants describe that PGD symptoms develop because the deceased was a next of kin. In contrast to “emotional bond,” participants emphasized the kinship relation and the deceased's role in the family more than the quality of the relationship.

*It's depending on the relationship. If the dead person is [someone] close and plays an important role in the family, there will be […] long lasting grief in the family. If the dead person has no significance and plays a little role in the family, grief or mourning may not stay long. (man 9, Ethopia)*

This cause was also mentioned more often by refugees from Arab countries. Participants described strong family bonds, also in the important extended family.

*Second, I would say the bond between family members in Arab families is so…there is this bond that they cannot leave each other. Or it is important that the whole family is there. (woman 4, Syria)*

###### Lack of Social Support (N = 10).

Almost half of all participants perceived the cause of PGD symptoms in insufficient support by family, friends, or the community. They either described that support as unavailable to help the person manage the loss or that the available support is not sought by the bereaved.

*Okay, so for example, someone is not married or has no close family, their grief is longer. That could be a cause. (man 5, Syria)*

*When you lose someone close and you don't have any other friends or so who you are as close with. Yes, I believe people with many friends and relatives grieve less. (woman 7, Yemen)*

Some participants specifically described greater loneliness and less support in Germany in comparison to their home country.

…*because at home you are not alone. You have family, you have brothers, sisters, cousins, the village community or friends who support you. But here, this loneliness, this being alone. (man 10, Ivory Coast)*

###### Material Dependency (N = 4).

The category “material dependency” was assigned when participants indicated that PGD symptoms develop because the relationship to the deceased was characterized by a dependency on their resources. They described, for example, a financial dependency and how losing the person leads to a crisis and difficulties to uphold one's life. For widows, this also includes a role reversal when they suddenly need to provide for their family.

*Because the man has a special role in our families. Not when it comes to raising children, that is a women's responsibility. But everything else, such as going to work, I mean making MONEY and EVERYTHING is done by the man. And now [when he dies] it is a burden on the women, because she must take over his role. (woman 6, Yemen)*

More statements regarding the importance of material dependency were made in the Sub-Saharan sample. Participants also described the value of a successful family member, irrespective of gender.

*So when the person is a breadwinner in the family, the person is supportive to everyone in the family. Let's say my mom is the only person that takes care of the family, providing us with food and shelter. And if we lost her, we would grieve for so many years to keep her in mind, because we can never and ever find someone like her again. (woman 10, Ghana)*

###### Aggravation by Others (N = 1).

One person from Iraq assumed that unhelpful social interactions intensify the grief reaction and cause avoidance symptoms described in the vignette.

*Or as I said, sometimes the atmosphere in a family also plays a role. When the mother of that man in the example, maybe in Iraq or somewhere, is also grieving and when he calls, she talks about it AGAIN and cries, this can also be a cause. (man 7, Iraq)*

##### Intrapersonal Causes

Causes for PGD symptoms that lie within the person were mentioned by 69.6% of all participants.

###### Vulnerability (N = 12).

The category “vulnerability” was assigned when participants expressed that PGD symptoms are caused by a predisposition in the person's personality or an acquired predisposition during their upbringing. Participants often described being too emotional or not having enough strength to cope with loss.

*There are strong people, but there are also weak people and everyone copes depending on their condition. That's why it is quicker for some and prolonged for others. There are people who by nature cannot deal with pain. Maybe that is a general cause. (woman 12, Cameroon)*

###### Guilt (N = 7).

Seven participants explained how they see pervasive feelings of guilt as a cause for PGD symptoms.

*She is probably feeling guilty, more or less because she thinks that she did not help and questions why this happened to her mother. (woman 5, Egypt)*

Guilt was most often mentioned in context of being separated from the family due to the flight. Being unable to care for them before they died was perceived as a strong source of guilt. This explanation was more common among Arab participants.

*I believe that she feels guilty because she was far away from her mother for a long time. For me, that would be a reason when my mother died now to be sad all the time because I left HER and left her alone. That would kill me. I would think about her forever and think “I should have come back or I should have just stayed.” (woman 6, Yemen)*

###### Pre-existing Mental Health Problems or Traumatic Experiences (N = 3).

Three participants indicated that a mental health condition that existed before the loss or previous traumatic experiences are causes of PGD symptoms.

*But when people were sad before or always depressed and then, on top, someone dies, then [the grief] will be prolonged. (woman 6, Yemen)*

*That requires so many aspects of trauma that someone withdraws like that…otherwise I could not explain it. Eritrea is a traumatized country because of the war and even people who can flee have experienced bad things. Women [have experienced] a lot of abuse, rape, scenes of murder and so on. (woman 9, Eritrea)*

###### Religious Beliefs (N = 1).

One participant from Syria referred to ruminating about God's reasons behind the death as a cause of PGD. This person emphasized how religion is interwoven with nearly all aspects of life.

*In Syria, everything is connected to religion. No matter what happens, God planned it for us; no matter what happens, God will save us or punish us. […] And if you are religious, [grief] is prolonged, because you think “Why did this happen? Why not me? What did I do to God? Did I pray enough?” And so on and so on and then it takes a long time and then you become depressed and you are sad. It is so prolonged because you have so many questions and don't get answers. (woman 4, Syria)*

##### Circumstances of the Death

Causes that are related to the circumstances of the death were mentioned by 65.2% of the sample and were grouped into five subordinate categories.

###### Violent or Unnatural Death (N = 8).

Eight participants perceived violent or unnatural circumstances of the death as the cause for PGD symptoms. They either referred to the assault described in the vignette or described war-related circumstances of loss in general. The impact of a violent or unnatural death was often compared to loss after a longer disease or a sudden condition like a heart attack.

*The circumstances of the death. If your mother dies after a long illness and in her bed in peace it is different than when she is assaulted and stabbed to death with a knife. That is something you cannot understand and think “My mother was a good person, why did someone something like that to her?” In my opinion, that is how someone could get prolonged grief disorder […]. (woman 12, Cameroon)*

Participants from Arab countries, especially Syrian participants, mentioned traumatic loss more often. They described how people die under atrocious circumstances (i.e., torture) and that seeing or knowing how much the loved one has suffered intensifies grief.

*So imagine a mother who for years sacrifices her life and her resources for her child. And then this child dies, yes this happens often that a bomb drops, and a splinter kills somebody. And this does not feel real anymore, how can this happen that THIS child was hit by a bomb. And unfortunately, it is also common that young people and elders are imprisoned and they are tortured and then two or three months later, [their body] will be passed on to their parents and you see… Well, they say “Was hit by a car” but you know this is not the truth, you can tell from the body how much the person suffered. And it is EVEN HARDER when the body is not returned, but just disappears. You can really grieve when you know someone is dead. (man 3, Syria)*

###### Sudden Death (N = 7).

Seven participants expressed that they see a sudden, unexpected death as the cause of PGD. Statements in this category emphasized the suddenness of the news of the death more than the circumstances. Again, more statements were made by participants from Arab countries.

*It is a possible cause—I mean in general and probably in this example—that you do not expect that the father …It is so sudden, a phone call and they say “Your father is dead.” And he thought that this is 20 or 30 years away. And maybe this shock does something. (man 7, Iraq)*

##### Unable to Perform Rituals (N = 6)

The category “unable to perform rituals” comprises statements regarding the importance of rituals. Participants described religious or spiritual rituals as well as sharing grief in the community in a ritualized way. Being unable to perform or participate in rituals, such as the funeral, due to the flight or living in a country where rituals are not possible, was perceived as the cause of PGD symptoms.

*It is even more painful when you can't be there for the funeral. There are rituals related to the funeral that need to be done. For example, as a Muslim you would wash the body and dig the grave and walk all the way from your home or the mosque to the graveyard. Some people drive but many do walk. It's these rituals [the man in the vignette] is missing. (man 1, Syria)*

Rituals were more often mentioned by participants from Sub-Sahara Africa who described rituals surrounding the funeral and the wake and how painful it is not being able to participate.

*He would have liked to be there that day [of the funeral]. I told you that we give a message to the deceased before we close the coffin. People from the family, even people from the village community come to the coffin, everyone brings their message: “If you meet my father, tell him I have problems here. Tell him, he needs to see my problems and help me.” And they will give their message to the deceased. And when you are far away and not part of the ceremony, that hurts. I would have liked to be there to tell my mother and father “That is my problem, my situation” so that it can get easier in Germany. And that causes additional grief, right? […] This tribe, this people don't only see the deceased as a deceased, it is not only a goodbye but also a transformation into a different state. The person hears and SEES what happens to the bereaved. They can help solve our problems and improve our situation and I would like to be there to give my problems to the person so that they can help solve the problems when they get to next level. And I cannot do that when I am 10.000 km away and that is additional grief, a potentiation of my grief. (man 10, Ivory Coast)*

###### Loved One Died Alone (N = 1).

One participant from Syria believed that PGD symptoms developed because the person was not with the loved one when they died.

*She could not be there [when her mother died]. I believe it can be more difficult compared to when you are with the person who is dying. Yes, I believe that is most difficult. (woman 2, Syria)*

###### Multiple Losses (N = 1).

Another participant from Syria suggested that PGD symptoms develop when more than one loss occurs within a short period of time.

*In some cases, like with my aunt, she was very happy before [her husband and her mother died] and behaved normally and that it was so extreme all of a sudden is just because she lost TWO people at once. (woman 6, Yemen)*

##### Separation From Home and Loved Ones (N = 7)

According to seven participants, PGD symptoms are caused by being far away from the home country due to the flight or migration.

*And regarding causes, first, I would say it is the long distance between the home country and Germany. (woman 2, Syria)*

Statements also include the separation from the family and participants explained that grief can become prolonged when someone had not seen the deceased for a long time before they died.

*When you have not seen each other in a long time. For example, someone who came to Germany to go to university and has not seen their parents in three or four years. And then the parents die. The person will not [get over it] … That could be a cause, a general cause. That is connected to grief, not having seen the parents in a long time. (woman 12, Cameroon)*

#### IPQ

In the questionnaire survey (see Table 3), participants endorsed “one's emotional state” (95.7%, *n* = 22), “stress or worry” (82.6%, *n* = 19), and “one's mental attitude” (82.6%, *n* = 19) as predominant causes behind PGD symptoms. Participants from Arab countries also attributed symptoms to “one's personality” (92.9%, *n* =13) and “family problems or worries” (92.9%, *n* =13). Agreement with religious and supernatural causes was markedly lower: 39.1% (*n* = 9) of the sample agreed with “God's will” (Arab sample: 42.9%, Sub-Saharan African sample: 33.3%), 17.4% (*n* = 4) with “evil spirits” (Arab sample: 7.1%, Sub-Saharan African sample: 33.3%), and 13% (*n* = 3) with “supernatural forces” (Arab sample: 14.3%, Sub-Saharan African sample: 11.1%).

**Table 3:** Causes of PGD symptoms assessed with the Revised Illness Perception Questionnaire (IPQ-R).

	**Total sample (*****N*** **=** **23)**		**Participants from Arab**		**Participants from Sub-Sahara**	
			**countries (*****n*** **=** **14)**		**Africa (*****n*** **=** **9)**	
**IPQ-R Cause**	***N***	**%**	***N***	**%**	***N***	**%**
Stress or worry	19	82.6	12	85.7	7	77.8
Hereditary	7	30.4	4	28.6	3	33.3
Germs or virus	1	4.3	0	0.0	1	11.1
Diet or eating habits	8	34.8	5	35.7	3	33.3
God's will	9	39.1	6	42.9	3	33.3
Chance or bad luck	4	17.4	2	14.3	2	22.2
Poor medical care	8	34.8	5	35.7	3	33.3
Environmental pollution	2	8.7	1	7.1	1	11.1
One's own behavior	17	73.9	10	71.4	7	77.8
One's mental attitude	19	82.6	12	85.7	7	77.8
Family problems or worries	18	78.3	13	92.9	5	55.6
Evil spirits	4	17.4	1	7.1	3	33.3
Overwork	14	60.9	11	78.6	3	33.3
One's emotional state	22	95.7	14	100	8	88.9
Aging	12	52.2	9	64.3	3	33.3
Alcohol	8	34.8	5	35.7	3	33.3
Smoking	6	26.1	3	21.4	3	33.3
Supernatural forces	3	13.0	2	14.3	1	11.1
Accident or injury	11	47.8	7	50.0	4	44.4
One's personality	18	78.3	13	92.9	5	55.6
Altered immunity	7	30.4	6	42.9	1	11.1

*PGD, prolonged grief disorder; IPQ-R, Revised Illness Perception Questionnaire*.

### Cures

Participants suggested different cures or treatments for the person described in the vignette and for people suffering from PGD in general. Cures were grouped into four superordinate categories: (1) interpersonal and professional help, (2) individual behaviors, (3) religion and spirituality, and (4) other cures (Table 4). Obstacles to treatment and healing were also discussed.

**Table 4:** Cures of PGD symptoms.

	**Total sample (*****N*** **=** **23)**		**Participants from Arab**		**Participants from Sub-Sahara**	
			**countries (*****n*** **=** **14)**		**Africa (*****n*** **=** **9)**	
**Cure**	***N***	**%**	***N***	**%**	***N***	**%**
**Religion/spirituality**						
Belief in God/spiritual beliefs	10	43.5	8	57.1	2	22.2
Belief in life after death	4	17.4	3	21.4	1	11.1
Accept the loss as God's will	3	13.0	3	21.4	0	0.0
**Interpersonal or professional help**						
Social support	18	78.3	12	85.7	6	66.7
Mental health professionals	12	52.2	4	28.6	8	88.9
Find someone to take the place of the deceased	3	13.0	3	21.4	0	0.0
Priests/elders	2	8.7	0	0.0	2	22.2
Support groups	2	8.7	2	14.3	0	0.0
**Individual behaviors**						
Focus on the present and pursue goals	7	30.4	6	42.9	1	11.1
Recognize and express painful emotions	6	26.1	5	35.7	1	11.1
Remember the deceased	5	21.7	4	28.6	1	11.1
Overcome guilt/think differently	3	13.0	2	14.3	1	11.1
Live like the deceased would have wanted you to do	3	13.0	3	21.4	0	0.0
Complete rituals	2	8.7	1	7.1	1	11.1
Avoid reminders	1	4.3	1	7.1	0	0.0
**Other cures**						
Time heals	2	8.7	1	7.1	1	11.1
Understand the reasons for one's reactions	1	4.3	0	0.0	1	11.1

*PGD, prolonged grief disorder*.

#### Interpersonal and Professional Help

The majority of participants (87.0%) regarded interpersonal or professional help as the most effective way to overcome PGD symptoms. Four subordinate categories emerged.

##### Social Support (N = 18)

Support from other people was perceived as most important in order to help someone with PGD symptoms. Participants explained that others can help by simply listening or providing an opportunity to share grief, but also by providing distraction and support in daily life.

*Just talk, social connection especially to people who have been friends before and family, that can help. Everything that has to do with social connection. In the Mediterranean culture, I believe people are more sociable or socially oriented. For example, I have never heard that somebody who was bereaved said “I don't want anyone here,” or something like that, I have NEVER heard that. It COULD happen, but that is an isolated case. (man 5, Syria)*

Support by family and friends was mentioned most often. Participants explained how valuable help from friends with similar experiences can be and emphasized the importance of a strong bond, trust, and understanding.

*The only help that can be given to the person is when somebody is there to talk to her. Somebody just sitting down, talk to her, advise her and help her and if she needs any help, the person is ready to support her. […] That is the only way to let the person forget about the dead mother. Because if no one is there to talk to her or to bring the person down or to support the person like the mother did before she died, she will be disturbed emotionally […]. (woman 10, Ghana)*

Participants also described the importance of offering support to others in the family or community. They explained how supporting the bereaved is also part of rituals and their culture.

*Sometimes I invite people when we have an event in our community. Something cultural, something educational, or sometimes we just meet to EAT together or something like that. And I look for people who are psychologically distressed by their grief so that they can mingle and don't have to stay so sad. (man 7, Iraq)*

The social withdrawal described in the vignette was perceived as concerning and strange by a few participants. One participant explained how this behavior stood in sharp contrast to what he is familiar with:

*I think it's weird that he avoided contact with his siblings because normally with us, in Syria, you try and get in touch with more people who were close to the bereaved. So for example, there are three, four, or five siblings and all of a sudden, the mother dies. Then these five siblings try to be even closer to each other so that they don't feel that the mother is suddenly gone. That was strange for me that the person did not want to be in touch with people who were close to the father. I believe this is how you could treat him. You could treat him after the death with bringing him closer to people who were also close with the deceased. (man 1, Syria)*

##### Mental Health Professionals (N = 12)

About half of all participants mentioned that seeking help from therapists, psychologists, or psychiatrists is important and were positive that these mental health professionals can help someone who suffers from PGD symptoms.

*Okay. The professional people they can help, because they are trained to do that. So they can help, they can talk to them, they can advise them and they know how it's affecting them emotionally and help them to forget about that. So I think the professionals, they can really, really help. Yeah. (woman 10, Ghana)*

*Yes, [my aunt] also realized she is different and she needed help and then she expressed that and went to see a doctor, a psychologist. (woman 6, Yemen)*

Some Sub-Saharan participants agreed that therapists can be helpful when probed by the interviewer, but generally professional help was regarded only as an option when social support or religion did not help.

*When somebody does not have a circle of friends, someone who says “You are like me. I also lost my father but look at me, I am still living” or “Do what I do,” when someone does not have this opportunity then psychologists or therapists or whoever are needed to support them. (man 10, Ivory Coast)*

*Then you can only look for help in therapy. […] If the person has prolonged grief despite having attended the funeral, he cannot do it on his own or others don't understand him. This condition is difficult. He needs someone to talk to, a therapy, a talking therapy. He needs someone who listens […]. He really needs help.[…] (woman 12, Cameroon)*

Participants also emphasized the differences between mental health services in their home country and in Germany. Some explained that they would seek help from therapists in Western host countries, but not in their home country.

*In Western cultures [mental health services] are advisable (laughs). I would recommend or at least say “Okay, talk to someone.” (woman 9, Eritrea)*

##### Find Someone to Take the Place of the Deceased (N = 3)

Participants from Arab countries mentioned that finding someone who takes the place of the deceased might cure PGD symptoms. They described that this can either be someone in the family who takes over the role of the deceased (e.g., mother takes over the role of the father) or someone else who becomes the new best friend. One participant also rejected this idea.

*Or [finding] a substitute for the deceased, but maybe that's out of the question. Some people try that, it happens in our culture, that people try to comfort you and say “Yes, you lost your brother but you have three other brothers.” For me, that was always a strange method because one person cannot replace another. But sometimes people try that. (woman 3, Syria)*

##### Support Groups (N = 2)

Two participants from Arab countries suggested seeking help from support groups. These groups can either be official or unofficial with the defining aspect that people who had similar experiences support each other. Because of the shared experiences as the main characteristic, these groups go beyond social support described above.

*Maybe by connecting to other people who are in the same situation, for example people who also lost someone due to an assault, and by talking about it together and everyone shares how they cope and everyone tries it until they have arrived in their daily life again. (woman 5, Egypt)*

One person explained that support groups often remain unofficial because of stigma but that this kind of structured support exists nonetheless.

*Support groups, but they don't exist under the name of support groups. People like to do that VERY much, very much for many many topics. It's really like that, people WANT to help but they don't say that they are a support group, because no one has the COURAGE to say “We are a support group for people with this specific disorder” because no one wants to disclose having this disorder. But as soon as someone says in a meeting or among friends “I have this problem,” people come and say “Yes, I also experienced that and my neighbors and my aunt they did this and that and that helped.” (woman 3, Syria)*

#### Individual Behaviors

When discussing cures, many participants (69.6%) described different individual behaviors that were grouped into seven subordinate categories.

##### Focus on the Present and Pursue Goals (N = 7)

The category “focus on the present and pursue goals” comprises statements regarding how the bereaved should focus on what they can do in the present or future rather than dwell on the loss. Participants gave examples of fulfilling activities of daily life but also mentioned pursuing long-term goals. They regarded this way of living as a possibility to take the person's mind off the loss that stands in contrast to withdrawal. More statements in this category came from participants from Arab countries.

*If someone has a hobby, they can do more of that. For example, some people write or paint. (woman 1, Syria)*

*Commit to a specific goal in your life. He will continue his life, it will not stop, he will live his life but with a goal. Without a goal, it's nothing. (woman 2, Syria)*

##### Recognize and Express Painful Emotions (N = 6)

Six participants explained that they see a cure for PGD symptoms in becoming aware of grief and other negative emotions and finding a way to express them. Participants also described cultural differences regarding the expression of emotions. A woman from Eritrea explained that it is common in her home country to share and express emotions and she perceived this as healing. A participant from Syria explained different expectations regarding the expression of emotions depending on the gender of the bereaved. While it is expected from women to show their emotions, the opposite is true for men which may hinder their coping with loss. More statements were made by participants from Arab countries.

*We have a proverb, it says “Crying or tears clean your soul.” And that's why we think it's good to be able to cry or something like that. (man 7, Iraq)*

*Just talking? Talking is the key to everything, for me personally, the key to everything. Open communication, where you express your emotions and cry. In the example, it's a woman, right? When we refer to men, I would say in the Arab culture, it's not okay that men cry. I would say it should be normalized that men cry and talk about their emotions because it is normal, it's because we are humans and not men and women. (woman 4, Syria)*

##### Remember the Deceased (N = 5)

Actively bringing up memories of the deceased, talking about them, and engaging in activities that remind of them was emphasized by five participants as being helpful in overcoming PGD symptoms. More statements were made by participants from Arab countries.

*For Syria, I would say when you lost someone you bring up memories more rather than keeping them at bay. People like it VERY much to talk about things they did with the deceased, what connected them, how strong their bond was, which emotions they still have related to the person, and sometimes that helps. If you don't do for too long, I would say it is helpful. If you occupy yourself INTENSELY with the topic for some time, then [grief] lessens and then you can find your way back into daily life. (woman 3, Syria)*

##### Overcome Guilt/Think Differently (N = 3)

Finding a new way of thinking about the loss was perceived as helpful by three participants. With reference to the vignette, they especially mentioned overcoming guilt.

*The example was about how the young woman blames herself, she had feelings of guilt. If she only forgave herself that she could not be there, that would be an immense help. To admit to herself, “Okay, I could not be there, I forgive myself.” (man 1, Syria)*

##### Live Like the Deceased Would Have Wanted You to Do (N = 3)

Three participants from Arab countries described living one's life in a way that would make the deceased happy as helpful. They gave examples such as choosing a career path that the deceased would have approved of or not being weighed down by grief too long but investing in one's future.

*When someone was very important to you then I believe it is very effective when you say “This person would not have wanted me to throw my life away for nothing but to continue living and invest in my life.” (man 3, Syria)*

##### Complete Rituals (N = 2)

One participant from each group perceived it as helpful when the bereaved completes important rituals associated with death, such as visiting the grave, or grieving with the community. This might also mean that someone must return to their home country.

*One cure would be to send the person back to his home country so that he can visit the grave. That would be painful, but that would give him peace, […] calm his thoughts. It will change something, will make something go away, you feel that, that difference. That's what it's like when you live in different countries. (woman 12, Cameroon)*

##### Avoid Reminders (N = 1)

One participant from the Arab group discussed that reminders that are too painful should be avoided in order to overcome PGD symptoms. She was drawing on experiences with a family member.

*She wanted to avoid the contact that always brought up painful memories or that reminded her of the person who died and that helped her. And that's why we needed to pay attention when my aunt was there […] that we do not talk SO much about my grandmother like “Grandma was like that and she did this.” No, we did not do this because it's not worth it. She died and it brings only grief and my aunt would be very sad. (woman 6, Yemen)*

#### Religion and Spirituality

Seeking help in religion or spirituality was perceived as helpful by 53.3% of all participants. Four subcategories emerged.

##### Belief in God/Spiritual Beliefs (N = 10)

A belief in God or spiritual powers was perceived to be of great importance. Participants explained how a strong belief, praying, or observing rituals may be the best help when someone is suffering from PGD symptoms.

*I can imagine that religion or a belief in God would make it easier for me. I used to work a lot with refugees and we had a program with a lot of psychologists and people told their stories how they came to Germany and how they lost people during their flight. There were extreme cases like one woman who said “I have lost my CHILD, I have SEEN how he died” and she can NEVER forget. Only the belief in God helped her. (woman 6, Yemen)*

The belief in God was more often mentioned by participants from Arab countries who were all Muslim.

*In my opinion, when you want to help people who are suffering from that, when you are working with Muslims, you should take a religious approach. (man 4, Syria)*

Some explained that the loss can be seen as a test from God that one needs to pass and that religious beliefs can motivate the bereaved to overcome grief.

*And the religion says that people who cope better and who have more patience in such cases will be rewarded by God. And this motivates you to keep it together and not break down when someone dies now. (woman 3, Syria)*

##### Belief in Life After Death (N = 4)

The category “belief in life after death” comprises statements regarding the belief that it will be possible to see the deceased again in the afterlife and that the deceased is living a better life now. Participants perceived these beliefs can be specifically helpful when someone is suffering from PGD symptoms.

*And then you also believe that the person who died, when it was wrongful that they were killed, then you believe this person is some kind of martyr and has a special place with God. And that's why you should not grieve so much because the person went to a better place. This also exists in Christianity, that you go to heaven and that you are in a better place. (man 3, Syria)*

*How you deal with the dead also depends on the upbringing and the culture you were born into. We Africans, we live with the dead. For us, the dead are not gone. They are not here anymore, we cannot touch them anymore, but they are present. That's why our original religion is this worship of ancestors. We pray to our ancestors, to those who have already died, who are with GOD and who can help US. And if you know that, that heals grief. You know, the deceased goes to the ancestors, to the grandparents and those who have died will built a community. That CURES, yes. (man 10, Ivory Coast)*

##### Accept the Loss as God's Will (N = 3)

Three participants from Arab countries emphasized that accepting the death as something that was wanted by God can help to cure PGD symptoms. These statements were related to the belief in life after death, but more tied to acceptance of the loss in the here and now than to the afterlife.

*And sometimes the sermon in the mosque. It helps when they say you have to be patient and you have to accept because everything came from God and then you have to accept what it is. They say that in the mosque. (man 7, Iraq)*

##### Priests and Elders (N = 2)

Two participants from Sub-Sahara Africa mentioned that seeking support from religious authorities as the most important treatment.

*In Africa, there are no jobs for psychologists. Psychologists in Africa are the priests, the pastors. We are also lawyers [laughs], this and that, and psychologists. When the person does not belong to an institutionalized religion, when he is an animist, that means traditional, there are two options. […] A psychologist cannot help a traditional person. […] If he is caught in this feeling, only older people who have grown up in this tradition can talk to him and show him other ways of tradition. (man 11, Congo)*

*In the case of Ethiopia, priests and elders, right? They play very, very important roles. Are addressing such problems. Many social problems, many problems related to stress that are caused by losing someone beloved or other relatives. Such kind of problems are addressed by such persons and such institutions. Such institutions play very, very important roles as social therapy in Ethiopia. (man 9, Ethopia)*

#### Other Cures

Two other cures that could not be grouped into the categories described above were described by three participants (13%).

##### Time Heals (N = 2)

One participant from each group expected that PGD symptoms become less intense with time without additional help.

And of course you need time to cope. I am not saying time heals all wounds but maybe you learn how to deal with it. (woman 9, Eritrea)

##### Understand the Reasons for One's Reactions (N = 1)

One participant from Sub-Sahara Africa mentioned that it is important to understand the reason behind the person's intense grief and build the treatment on that.

*Everything happens for a reason […]. So when you know the reason, you can think about a solution. Then I think this solution can be the treatment for this person's situation. (woman 11, Somalia)*

#### Obstacles

When participants talked about potential cures, about half of them (52.2%) also mentioned obstacles they perceive in the treatment of someone with PGD.

##### Mental Health Stigma (N = 4)

Four participants talked openly about mental health stigma. They described that seeking support from mental health professionals or disclosing prolonged grief as a mental health problem can lead to being labeled as crazy or being regarded as an outsider. The anticipation of these reactions can prevent people from seeking help.

*It is not normal to see a psychologist because then the whole family will be judged as strange like “Oh, they are treated for psychological symptoms, they are crazy.” You don't see a psychologist and if you do then you don't talk about it. I know how people suffer who talk about it […]. They don't only say “I'm ILL,” they will also be regarded differently from the COMMUNITY and they need to accept that people will give them funny looks and talk behind their back and say “She is ill, she is crazy, she needs to take medication, that is not normal.” (woman 6, Yemen)*

*But in our society in general we have a problem with psychiatrists, for example, or with seeing a psychologist. It's like “Why should I, I am not a sick person,” it is not accepted, it really is a taboo. I cannot image that I go to someone who has prolonged grief disorder in Syria and tell them “You need to see a psychologist.” Then they say “I am only grieving and how can you recommend something like that or imply I am mentally ill?” That doesn't work and one should not try it. (woman 3, Syria)*

##### Bereaved Person Has to Want Help (N = 4)

Participants from Arab countries emphasized that PGD symptoms can be cured but only if the person is willing to accept help.

*When he would accept help, then yes, [symptoms could be cured]. If not, the best therapy cannot help him. (man 2, Syria)*

##### Old Age (N = 3)

Three participants from Arab countries perceived it as more difficult to help someone with PGD in older age. They described that behaviors, thoughts, and relationships of young people are easier to change and older people might have less resources.

*I would say for people who are older, over 50 or 60, it is not as easy as for younger people. They won't be as open and they won't accept everything because they will think “No. What we think is correct because we are grown up and know better than young people.” I can understand that [laughs]. It is difficult because I cannot imagine talking to an older woman saying “NO, you just have to accept that. And you have to forget what has happened and you have to make your children's lives easier.” She would not say “Okay.” So, I think that is not easy, […] especially when we talk about Arab families. (woman 4, Syria)*

##### Therapist Cannot Help (N = 3)

Three participants from Sub-Sahara Africa disclosed doubts regarding Western therapists. They specifically emphasized that in order to help someone, a therapist must understand the patient's culture and important traditions related to loss.

*How can I say it? I am a bit uncertain regarding these psychologists. I have not been in therapy in my life so far, but I know something from my friends who have been in therapy because they could not deal with situations on their own or because they had disorders. I know that. But how far can a psychologist go? There is a person who comes to them saying “I have just lost my father and I cannot manage.” For me, the psychologist needs to be familiar with the person's culture, the culture they grew up in, with the traditions regarding grief, and with their life and reality in Germany. And then the person can help, but when they only know from books, when they read something or learned something at university, I think it will be the wrong way. (man 10, Ivory Coast)*

One participant also described that therapists are only consulted by “severe cases” and that grief should be dealt with within the family.

*But in our society, there are no services for [someone with prolonged grief], there are no psychologists. Well, there are psychologists, but they are for the severe cases, not for someone who grieves. […] The psychologist will be the family, an uncle, an aunt or someone from the family who will try. They are no psychologist, they have no method, but they will try […] to elicit positive feelings. (woman 12, Cameroon)*

##### Close Relationship to the Deceased Person (N = 2)

Two participants expressed doubts about healing PGD when the relationship with the deceased was very close.

*As I said, I find this difficult… If the best friend died or is gone, I believe it is the most difficult thing to help someone. (man 1, Syria)*

## Discussion

The study combined semi-structured interviews with a questionnaire-based survey in order to complement Arab and Sub-Saharan African refugees' subjective beliefs about causes and cures of PGD with established constructs. In the qualitative part of the study, causes for PGD symptoms were either seen in interpersonal aspects, intrapersonal aspects, or factors associated with circumstances of the death or separation from loved ones. Most participants attributed PGD to a close familial or emotional relationship with the deceased, lack of social support, pre-existing vulnerability, or guilt related to the death. Flight-related aspects that include traumatic experiences and circumstances of the loss as well as the inability to care for loved ones and participate in rituals surrounding the death were perceived as particularly important. Participants in both groups did not express supernatural beliefs. Likewise, in the quantitative part of the study, participants attributed symptoms to psychological factors and rates of agreement for supernatural causes were much lower. All participants believed that PGD symptoms can be cured and named interpersonal and professional help, individual behaviors, and religion and spirituality as potential cures. In line with the belief about social causes, they were most inclined to expect help from family, friends, and social support in general. The majority of participants did not initially mention psychological treatments or even voiced concerns regarding stigmatization or being understood by Western therapists.

Although the study was not designed to elicit differences between the two cultural groups, some interesting tendencies emerged. Especially statements from Syrian participants made clear how sudden deaths under traumatic circumstances (e.g., bombings, torture) impact grief reactions and these statements were clearly shaped by the ongoing war in Syria. Consistent with the literature (55), Arab participants also put more emphasis on religion and the belief in God. The large difference regarding agreement with mental health services as a cure for PGD—88.9% of Sub-Saharan participants made statements regarding mental health professionals but only 28.6% of Arab participants—can be explained by the way the interviews were conducted. The person who interviewed the Sub-Saharan group asked about opinions regarding mental health professionals when participants did not make a statement on their own while no such follow-up questions were asked in the Arab group. Some Sub-Saharan participants agreed that therapists can be helpful, but generally professional help was regarded only as an option when social support or religion did not help.

Lay explanatory models in our study partly overlap with established risk factors and models for PGD. Previous research has shown that a perceived lack of social support is a risk factor and that PGD is more likely after the loss of a close relationship or a death under unnatural circumstances (25, 56). For refugees, the role of migration- or adaptation-related difficulties and lack of rituals in PGD has also been documented (19, 57). Dysfunctional cognitions, including guilt, also play a major role in the development and maintenance of PGD symptoms [e.g., (58)]. Although opinions about grief disorders and their expression have been investigated in different cultures [e.g., (20, 40, 41)], there are no studies on perceived causes of PGD. However, there are several studies on beliefs about the causes of mental health problems in general or PTSD or depression specifically in different cultural groups. Beliefs expressed in the study are also consistent with findings regarding lay beliefs about causes of PTSD among refugees (29), especially regarding the role of traumatic life experiences and social causes. However, in contrast to previous research that showed a preference for supernatural explanations especially in cultural groups from Africa [e.g., (30, 31), participants in the present study did not offer any supernatural explanations in the interviews and only few agreed in the questionnaire survey. While Grupp et al. (29) also found that supernatural explanations should not be overstated, this was even more pronounced in the present study. There are several explanations. First, participants in this study had lived in Germany for a mean duration of 8 years and it is possible that they had somewhat adopted Western perspectives or experienced that supernatural explanations are not shared by the majority society. Second, symptoms of PGD vary from normative grief reactions in intensity and duration, but they are generally more familiar than symptoms of depression or PTSD because normative grief is a universal experience. It therefore seems possible that people are less inclined to search for supernatural explanations.

Treatment expectations were overall directed at ameliorating the factors that were believed to have caused PGD symptoms. Hence, healing was expected from re-engaging with the family and the community. Increasing social support is indeed one element in some evidence-based treatments for PGD [e.g., (59)] and a focus on grief-related daily problems has also been found to be helpful (60). Participants also put much emphasis on religious or spiritual authorities and practices which is in line with previous findings regarding the role of religion in help-seeking of refugee groups [e.g., (28, 30, 33, 34)]. While participants described their belief as a source of comfort, it is important to note that reliance on religious authorities can also be a gatekeeper to seeking health care (31, 33, 34).

It is noteworthy that social support and religion were perceived as the first-line help in this study. Although some participants mentioned mental health professionals, it was clear that they had reservations and perceived barriers to their utilizations. We did not ask participants about their knowledge of the health care system and it is possible they were not familiar with available services and their methods as some statements as well as past research has indicated (34, 37). Mental health services are often not well-known by refugees because less mental health care is available in their countries of origin (38). Some participants also struggled with understanding what we mean by PGD and the interviewers needed to take time to disentangle beliefs about normative and disturbed grief. This may have been due to PGD being a new diagnosis that is not well-known yet, but other studies have also found that a lack of mental health literacy in general is a barrier to help seeking in refugee groups (61–63).

Participants also named mental health stigma as an obstacle to seeking help for PGD symptoms and mentioned fears of being ostracized from the community. Previous research has repeatedly shown that stigma is among the main barriers to seeking help for mental health problems (37, 64). It is also of importance that expectations from mental health professionals were low because some participants did not believe that a Western therapist could understand them. This concern was also voiced by Sub-Saharan asylum seekers regarding the treatment of PTSD (34) and problems with trust were previously reported by mental health providers (63). Together with treatment expectations, these perceived obstacles have implications for the adaptations of interventions for refugees suffering from PGD.

### Implications for Cultural Adaptation

Our findings support the need for a culturally sensitive approach to PGD treatment when working with refugees. Three aspects seem important in order to reach refugees with PGD with psychological interventions: (1) overcome barriers to help-seeking, (2) establish a shared explanatory model, and (3) adapt treatment components and delivery.

Psychological interventions for PGD are successful (24), but therapy was not among the first choices for participants in our study. In order to prevent a large treatment gap for PGD, barriers to help-seeking need to be overcome. First, it seems important to reduce stigma through low-threshold interventions or campaigns, preferably supported by members of the respective cultural groups. A short intervention specifically directed at reducing stigma has been shown to be successful (65). There is also a need for education about PGD as a mental health problem and available services. This can be done with general intervention programs aimed at newly arrived refugees [e.g., (66)] or interventions that increase engagement with therapy (67). Working with religious authorities may be a way to prevent them from becoming gate keepers. Therapists also need to take concerns regarding trust and being understood seriously. As the numbers of patients from diverse cultural groups can be expected to rise in the future, training for therapists needs to focus on delivering treatment in a culture-sensitive way and therapists from the majority society need to make an effort to understand their patients' experiences, traditions, and expectations.

Evidence-based treatments are based on their specific models for the development and maintenance of PGD. In order to engage patients in therapy, models should be adapted to patients' beliefs insofar that patients share the rationale behind the treatment (21). When treating refugees with PGD, individual models can include and educate about established factors of PGD development (e.g., avoidance behavior) and combine them with a patient's individual important aspects. This means that, for example, for patients from collectivistic societies, the role of support by family and community can be included in a model. Beliefs expressed by participants in the present study also underline the importance of addressing flight and post-migration-related aspects. Therapists should, for example, ask about the circumstances of the loss and their meaning. If not offered by the patients, therapists should also inquire about traditions and rituals surrounding a death and if they could be completed.

As adapted interventions are more effective than non-adapted versions of the same intervention (68), treatment components and their delivery also need to be adapted (21). As some treatment components of evidence-based intervention programs matched our participants' expectations, these programs seem well-suited for the treatment of refugees. Combining them with culture-specific rituals and religious dimensions and tailoring delivery and materials to patients' need can result in a culturally sensitive treatment.

Rituals can be integrated into therapy (69). If they cannot be completed in real life, it may also be possible to use mental imagery that can have a powerful impact resembling real-life experiences (70). If religious practices or authorities are important for a patient, they can be consulted or also incorporated into therapy. Furthermore, findings regarding the importance of social support point toward group settings as an option for treatment delivery. The needs of patients from predominately collectivistic cultures could be met by enhancing mutual support and at the same time it would be possible enroll more patients in a shorter period of time, thus significantly reducing waiting times for therapy.

### Limitations and Implications for Future Research

Despite some valuable insights, the study has number of limitations. First, the small sample size does not allow generalization and especially the Sub-Saharan African sample and non-Syrian Arab sample were very heterogeneous regarding countries of origin. Potential differences between the cultural groups included in the study and beyond need to be examined in future studies. Second, while the vignette allowed for a specific presentation of PGD symptoms, it is also possible that open interviews would have generated slightly different responses. As we did not include a comparison group without a migration background, we cannot infer that refugees' beliefs differ from those held by German residents. Future studies that conduct direct comparisons between larger refugee and non-refugee groups are therefore needed. Third, data collection took part at during the first weeks of the COVID-19 pandemic in Germany when regulations for physical distancing were in place. Interviews were therefore conducted online which made working with interpreters more difficult. Interviews were mostly conducted in German or English which constitutes a selection bias. A slight language barrier still remained in some interviews. This was more often the case for Sub-Saharan African participants and could explain why more ideas were offered by Arab participants. Conducting interviews in the participants' native language could therefore lead to deeper insights in future studies. COVID-19-related difficulties also hampered recruitment and we interviewed all participants who volunteered during the specific time period and did not continue until data saturation was reached. Last, interviews were conducted by white females without a migration background. Although participants expressed that they find the study valuable and were willing to explain rituals and cultural perceptions in detail, their responses may have been shaped by social desirability.

### Conclusion

Our findings suggest that mental health professionals should explore and understand the relationship with the deceased and circumstances of the loss in conflict regions as well as important rituals when working with refugees with PGD. At the same time, cultural differences should not be overstated, because beliefs about causes showed similarities with Western conceptualizations. The expressed treatment expectations underline the importance of reducing stigma surrounding mental health care and increasing knowledge. With some adaptations, available PGD treatments have the potential to also meet refugees' needs. Future studies on different cultural groups and culture-sensitive treatments for PGD are needed.

## Data Availability Statement

The dataset generated for this study can be found in the Supplementary Material. The qualitative data presented in this article are not readily available to protect the privacy of the participants. Reasonable requests to access the data should be directed to the first author.

## Ethics Statement

The studies involving human participants were reviewed and approved by Ethics Committee of the Goethe University Frankfurt, Fachbereich 05. The patients/participants provided their written informed consent to participate in this study.

## Author Contributions

FL-M and HC developed the study design, supervised the data collection, conducted the analyses, and wrote the manuscript. All authors contributed to the article and approved the submitted version.

## Funding

The study was funded by Freunde und Förderer der Goethe Universität Frankfurt [Friends and Sponsors of the Goethe University Frankfurt].

## Conflict of Interest

The authors declare that the research was conducted in the absence of any commercial or financial relationships that could be construed as a potential conflict of interest.

## Publisher's Note

All claims expressed in this article are solely those of the authors and do not necessarily represent those of their affiliated organizations, or those of the publisher, the editors and the reviewers. Any product that may be evaluated in this article, or claim that may be made by its manufacturer, is not guaranteed or endorsed by the publisher.

## Footnotes

^1^Descriptions are based on explanations by study participants.

^2^Quotes from interviews conducted in German were translated into English by the first author.

## Supplementary Material

Supplementary Data Sheet 1 Interview guideline.

Supplementary Data Sheet 2 Questionnaires.

Supplementary Data Sheet 3 COREQ checklist.

Supplementary Data Sheet 4 Survey data.
