# Supplementary material for: Beliefs About Causes and Cures of Prolonged Grief Disorder Among Arab and Sub-Saharan African Refugees
Source: Front Psychiatry. 2022 Apr 5;13:852714. doi: 10.3389/fpsyt.2022.852714 (PMC9037322; doi:10.3389/fpsyt.2022.852714)
Supplement: Supplementary Data Sheet 1 — Interview guideline. [file Data_Sheet_1.pdf]

frontiers

Supplementary Material

1 Semi-structured interview guideline

Introduction

Thank you for taking the time to participate in our study. Before we start, I would like to briefly introduce myself a little. As you already know, my name is ___. I live in __ and originally I from___/ my family lives in ____. I am currently studying for a Master's degree in psychology and will conduct this interview as part of my master's thesis.

It is our goal to learn more about the expression and beliefs of Prolonged Grief Disorder in Arab/Sub-Saharan African countries. Prolonged Grief Disorder is a mental disorder in which grief persists over along time and causes significant problems in daily life.

During our email conversation/phone call you already told me that you are from _____ (country). Please answer my questions below as if you were a representative of your country ___. Please take the time you need during the interview to think about the beliefs and opinions are in country _____.

Do you have any questions at this point? / Then we can start. I'm going to read an example of a person with Prolonged Grief Disorder out loud. I will also share my screen so that you can read it with me. Afterwards, I'm going to ask you a few questions related to this person.

a) Symptoms of disturbed grief

[not part of the present study]

b) Duration of grief

[not part of the present study]

c) Beliefs about causes

What do you think caused the described person’s prolonged and impairing grief?

(*explain if necessary:* Why does the person grieve so much and for such a long time compared to others? Why doesn’t their grief lessen over time?)

What do you think could cause Prolonged Grief Disorder in general?

d) Beliefs about cures

Do you think there is help for the person?

If yes: How can the person be helped?

If no: Why do you think there is no help for this person?

Do you think that prolonged grief disorder can be cured in general?

If yes: What could help?

If no: Why do you think there is no help?

Miscellaneous

Do you think men and women grieve differently? If yes, how?

What do you think has shaped your view of grief in general? (For example, you might have been influenced by your religion, the opinions within your family or friends, or the opinions in your culture).

End

Thank you very much. That is all for now. Is there anything else you would like to add that we have not addressed so far? Do you have any questions for me?

Once again, thank you very much for your time and sharing your views and experiences. After we have finished, I will send you a link via email and would like to ask you to fill out three more questionnaires.
