# Supplementary material for: Beliefs About Causes and Cures of Prolonged Grief Disorder Among Arab and Sub-Saharan African Refugees
Source: Front Psychiatry. 2022 Apr 5;13:852714. doi: 10.3389/fpsyt.2022.852714 (PMC9037322; doi:10.3389/fpsyt.2022.852714)
Supplement: Supplementary Data Sheet 2 — Questionnaires. [file Data_Sheet_2.pdf]

Supplementary Material

2 Questionnaires

Sociodemographic Questionnaire

1. How old are you?
   ________ years

2. What is your gender?

   ☐ Female

   ☐ Male

   ☐ Diverse

3. For how long have you lived in Germany? ___________ years

4. In which country did you live before you came to Germany?

   ____

Is this your country of birth?

☐ Yes ☐ No

If no, in which country have you been born? ______________________

5. If relevant to you, which ethnic group do you belong to?

   ____

6. What is your religion?

   ☐ Muslim

- ☐ Christian
- ☐ Jewish
- ☐ No religion
- ☐ Other religion: ___________

7. For how many years did you attend school? ___________ years
8. What is your highest degree? ___________________
9. Are you currently working?

- ☐ Yes, as ___________________
- ☐ No

Causes of prolonged grief

With this questionnaire we would like to learn about your personal beliefs regarding symptoms of persistent impairing grief and Prolonged Grief Disorder.

We are interested in what you consider may have been the cause of prolonged grief in the person you read about at the beginning of this survey. We are most interested in your own views about the factors that caused this disorder in the person described in the example. Below is a list of possible causes for this disorder. Please indicate how much you agree or disagree that they were causes for the person by ticking the appropriate box. Please answer all questions.

		Strongly disagree	Disagree	Neither agree nor disagree	Agree	Strongly agree
1	Stress or worry					
2	Hereditary – it runs in the family					
3	A germ or virus					
4	Diet or eating habits					
5	God’s will					
6	Chance or bad luck					
7	Poor medical care in the past					
8	Pollution in the environment					
9	The person’s own behavior					
10	Mental attitude of the person e.g. thinking about life negatively					

11	Family problems or worries caused the problem					
12	Evil spirits					
13	Overwork					
14	Emotional state of the person, e.g. feeling down, lonely, anxious, empty					
15	Ageing					
16	Alcohol					
17	Smoking					
18	Supernatural forces / curse					
19	Accident or injury					
20	Personality					
21	Altered immunity					

In the table below, please list in rank-order the three most important factors that you now believe caused prolonged grief in the person described in the example. You may use any of the items from the box above (1-21), or you may have additional ideas of your own.

The most important causes of the illness of the person described in the example:

1. ____________________________
2. ____________________________
3. ____________________________

Traumatic Grief Inventory- Self Report[1]

The following questionnaire contains two parts. In **Part 1**, you are asked about the losses of loved ones you have been confronted with. **Part 2** asks you to what extent you experience grief reactions related to the most distressing loss you have experienced.

Part 1

Please indicate whether or not you have experienced the death of the person mentioned. Be sure to consider your entire life (growing up as well as adulthood) as you go through the list of persons.

	**Person**	**Deceased**
**1.**	Partner	◯
**2.**	Child<br>If more than one child: How many children are deceased? _____	◯
**3.**	Mother	◯
**4.**	Father	◯
**5.**	Sibling<br>If more than one sibling: How many siblings are deceased? ____	◯
**6.**	Other relatives<br>If yes: How many are deceased? ____<br>If yes: What was the degree of relationship with the deceased person? __________	◯
**7.**	Friend	◯

[1] Paul Boelen, Geert Smid (2013). Traumatic Grief Inventory-Self Report Version (TGI-SR) – English version. Edition: Foundation Center '45, Arq Psychotrauma Expert Group, Diemen. / Part 1 was modified for the present study.

	If yes: How many are decreased? _______________<br><br>If yes: Since when were you friends? ____________	

Which loss is still most distressing for you?
Number.: _____

Please think about the loss that is still most distressing for you and answer the following questions about the circumstances of this loss.

When did the person die?	|_|_| months ago; or |_|_| years and |_|_| months ago
What gender did the person have?	O female O male O other
How old was the person?	|_|_| years
How did the person die?	O Disease<br><br>O Act of violence<br><br>O Traffic accident<br><br>O Other accident: _____________<br><br>O Suicide<br><br>O Other: ____________________

Part 2

In this part you are asked to do the following two things:

1. From the persons who died, listed in Part 1, please select one person whose death is currently mostly on your mind or is currently most distressing you. Write down the name of this person:

The loss that is currently mostly on my mind/distressing, is the death of:

____

2. Below, several grief-reactions are listed. Please indicate how often you have experienced each reaction **in the past month**, in response to the death of this person.

		Never	Seldomly	Sometimes	Frequently	All the time
**1.**	I had intrusive thoughts or images related to the person who died.					
**2.**	I experienced intense emotional pain, sadness, or pangs of grief.					
**3.**	I found myself longing or yearning for the person who died.					
**4.**	I experienced confusion about my role in life or a diminished sense of self.					
**5.**	I had trouble accepting the loss.					
**6.**	I avoided places, objects, or thoughts that reminded me that the person I lost has died.					
**7.**	It was hard for me to trust others.					
**8.**	I felt bitterness or anger related to his/her death.					
**9.**	I felt that that moving on (e.g., making new friends, pursuing new interests) was difficult for me.					
**10.**	I felt emotionally numb.					

**11.**	I felt that life is unfulfilling or meaningless without him/her.					
**12.**	I felt stunned, shocked, or dazed by his/her death.					
**13.**	I noticed significant reduction in social, occupational, or other important areas of functioning (e.g., domestic responsibilities) as a result of his/her death.					
**14.**	I had intrusive thoughts and images associated with the circumstances of his/her death.					
**15.**	I experienced difficulty with positive reminiscing about the lost person.					
**16.**	I had negative thoughts about myself in relation to the loss (e.g., thoughts about self-blame).					
**17.**	I had a desire to die in order to be with the deceased.					
**18.**	I felt alone or detached from other individuals.					
